# Toe brachial blood pressure measurement after 5, 10, and 15 minutes of rest

**DOI:** 10.1186/1757-1146-6-S1-O33

**Published:** 2013-05-31

**Authors:** Sean Sadler, Fiona Hawke, Jennifer Sonter, Vivienne Chuter

**Affiliations:** 1 Podiatry Department, The University of Newcastle, Ourimbah, NSW, 2258, Australia

## Background

Toe Brachial Index (TBI) is used to evaluate peripheral arterial status, yet the effect of the duration of pre-test rest on TBI has not previously been evaluated. We aimed to investigate the effects of 5, 10, and 15 minutes of pre-test rest on TBI, and inter-rater and test-retest reliability.

## Methods

Eighty participants (57.5% female, mean age 70 years) were recruited from local podiatry clinics and The University of Newcastle. Automated Systoe (Atys Medical) and MicroLife (BP A100 Plus) devices were used to measure toe and brachial systolic blood pressures respectively, after 5, 10, and 15 minutes of rest. Two Podiatrists measured 20 participants on the same day to establish inter-rater reliability. Test-retest reliability was assessed by a single Podiatrist in 33 participants over two sessions, seven days apart. Effect of amount of pre-test rest on TBI was evaluated in 80 participants by a single Podiatrist.

## Results

TBI inter-rater (ICC 0.71) and test-retest (ICC 0.77) reliability were highest after 15 and 10 minutes of pre-test rest respectively. There was a significant increase in TBI between 5 and 10 minutes of pre-test rest (0.032; 95% CI: 0.52 to 0.012; *p* <0.0001), however the decrease in TBI between 10 and 15 minutes (0.004; 95% CI: -0.023 to 0.015; *p* = 1.000) was not significant.

## Conclusion

Results of this study suggest 10 minutes of pre-test rest is most appropriate for performing a TBI. The establishment of an evidence-base for pre-test rest time may improve clinical utilisation of TBI measurements.

